# No time to die: improving response to emergency scenarios in the 136 suite

**DOI:** 10.1192/bjo.2021.554

**Published:** 2021-06-18

**Authors:** Rebecca McKnight, Nicola Combs

**Affiliations:** Greater Manchester Mental Health Trust

## Abstract

**Aims:**

Improve confidence and experience of trainees performing preliminary medical reviews in the 136 suite.

Improve patient safety by increasing trainee's confidence in responding to emergency scenarios, including crash calls of patients in the 136 Suite.

To orientate trainees to the 136 suite and the emergency crash equipment, in order to better prepare trainees for emergency scenarios.

**Background:**

The authors encountered a crash call in the 136 suite, in which a patient had concealed an opiate overdose. The patient was successfully resuscitated but concerns were raised by the junior doctors that they were unaware of what or where the emergency equipment was kept in the 136 suite. Following a debrief session, we established that junior doctors needed more orientation to the 136 suite and more teaching on performing preliminary medical reviews and responding to emergency situations.

**Method:**

Trainees, were asked to complete an anonymous, qualitative questionnaire with 16 questions asking about their confidence to respond to emergency situations in the 136 suite.

Based on the feedback, an interactive teaching session was delivered two weeks later. The session covered a structured approach on how to perform a preliminary medical review and scenario-based teaching on emergency situations. Trainees were then shown the 136 facility, introduced to the lead nurse and shown the emergency crash equipment and drugs stores.

Trainees were then re-consulted, with the same questionnaire to ascertain whether confidence and knowledge had increased.

**Result:**

Following initial induction, only 25% of trainees felt confident performing 136 Suite preliminary reviews. 50% of trainees had encountered crash calls at Park House Hospital, however 93% did not receive orientation of emergency equipment locations. Only 44% of trainees felt confident managing a crash call; reasons included feeling ‘rusty, little recent experience, not being familiar with the equipment’.

Post-interactive teaching session, 89% now felt confident performing 136 Suite preliminary reviews. 100% knew where the crash equipment was located in the 136 Suite.

**Conclusion:**

Trainees should receive a robust induction on how to perform 136 preliminary reviews and have orientation of the facility, including crash equipment during induction

Trainees require refresher training in addition to their basic life support training on common emergency scenarios encountered in psychiatric hospitals.

A resuscitation skills training session is being organised for new trainees and hopefully incorporated into each forthcoming rotation.

